# *EWSR1-BEND2* fusion defines an epigenetically distinct subtype of astroblastoma

**DOI:** 10.1007/s00401-021-02388-y

**Published:** 2021-11-25

**Authors:** Calixto-Hope G. Lucas, Rohit Gupta, Jasper Wu, Kathan Shah, Ajay Ravindranathan, Jairo Barreto, Melissa Gener, Kevin F. Ginn, Owen W. J. Prall, Huiling Xu, Damien Kee, Hyun S. Ko, Nausheen Yaqoob, Nida Zia, Adriana Florez, Soonmee Cha, Arie Perry, Jennifer L. Clarke, Susan M. Chang, Mitchel S. Berger, David A. Solomon

**Affiliations:** 1 Department of Pathology, University of California, San Francisco, 513 Parnassus Ave, Health Sciences West 451, San Francisco, CA 94143 USA; 2 Department of Pathology, Children’s Mercy Hospital, Kansas City, MO USA; 3 Department of Pediatric Hematology and Oncology, Children’s Mercy Hospital, Kansas City, MO USA; 4 Department of Pathology, Peter MacCallum Cancer Centre, University of Melbourne, Melbourne, VIC Australia; 5 Department of Medical Oncology, Peter MacCallum Cancer Centre, University of Melbourne, Melbourne, VIC Australia; 6 Department of Cancer Imaging, Peter MacCallum Cancer Centre, University of Melbourne, Melbourne, VIC Australia; 7 Department of Histopathology, Indus Hospital and Health Network, Karachi, Pakistan; 8 Department of Pediatric Hematology and Oncology, Indus Hospital and Health Network, Karachi, Pakistan; 9 Department of Pathology, Fundación Santafé de Bogotá, Bogota, Colombia; 10 Department of Radiology and Biomedical Imaging, University of California, San Francisco, San Francisco, CA USA; 11 Department of Neurological Surgery, University of California, San Francisco, San Francisco, CA USA; 12 Division of Neuro-Oncology, Department of Neurological Surgery, University of California, San Francisco, San Francisco, CA USA; 13 Department of Neurology, University of California, San Francisco, San Francisco, CA USA

Astroblastoma is a glial neoplasm previously diagnosed solely on the basis of histologic features including tumor cells with stout processes arranged in perivascular pseudorosettes (so-called “astroblastomatous” rosettes), often embedded within a sclerotic stroma [2, 3]. However, refinement in molecular-based tumor classification schemes has led to the realization that this histologic pattern is not specific to astroblastoma and can also be seen in other genetically defined tumor entities, including *BRAF*-mutant pleomorphic xanthoastrocytoma, *ZFTA*-fused supratentorial ependymoma, and *IDH*-wildtype glioblastoma [1, 7, 13]. A recent genomics study demonstrated that a large subset of tumors with astroblastoma histologic appearance harbor *MN1* gene fusion (typically with *BEND2* as the fusion partner) and resolve into a distinct epigenetic cluster, which was initially termed “high-grade neuroepithelial tumor, *MN1*-altered” [11]. Multiple subsequent studies have confirmed that *MN1* fusions define a group of circumscribed glial neoplasms with astroblastoma-like morphology (with or without a primitive embryonal-like component), female predominance, and a propensity for local recurrence, but with mostly favorable clinical outcomes [1, 7, 13]. The identification of *MN1* fusion by next-generation sequencing or *MN1* rearrangement by break-apart fluorescent in situ hybridization (FISH) can be used to support the diagnosis of “astroblastoma, *MN1*-altered” per recent cIMPACT-NOW recommendations [8], and this molecularly-defined tumor type has been adopted in the 5^th^ edition of the WHO Classification of Tumors of the Central Nervous System [4]. Of note, however, are a few recent case reports of gliomas with astroblastoma-like histology lacking *MN1* alterations and instead harboring *EWSR1-BEND2* fusion [9, 10, 12, 14]. The biologic relationship of these gliomas with *EWSR1-BEND2* fusion compared to the newly defined tumor type “astroblastoma, *MN1*-altered” is currently unresolved.

We performed targeted next-generation sequencing and genome-wide DNA methylation profiling on a cohort of four patients with *EWSR1-BEND2* fused gliomas. The one male and three females had a median age at time of initial diagnosis of 13 years (range 6–26 years) (Fig. 1a). Two patients had tumors located in the frontal lobe and presented with seizures, whereas the other two patients had tumors located in the brainstem or cervical spinal cord and presented with limb weakness. Imaging revealed enhancing mass lesions, sometimes associated with a cystic cavity (Fig. 1b, c; Supplementary Fig. 1 [Online Resource 1]). Extent of resection, adjuvant therapy, and clinical outcomes are listed in Supplementary Table 1 [Online Resource 2]. Histologically, these were circumscribed gliomas composed of epithelioid tumor cells embedded within a densely sclerotic stroma, with variably prominent astroblastomatous rosettes (Fig. 1d, e; Supplementary Fig. 2 [Online Resource 1]; Supplementary Table 2 [Online Resource 2]). A more primitive, embryonal-like component was prominent in two tumors and focally present in the other two. Mitotic activity was conspicuous (often > 5 mitoses per 2 mm^2^), and foci of necrosis were identified in all cases, but were not associated with peripheral palisading of tumor cells. No microvascular proliferation, Rosenthal fibers, or eosinophilic granular bodies were identified. Immunohistochemical evaluation revealed variable patchy expression of glial markers (GFAP and OLIG2), minimal to absent expression of neuronal markers (synaptophysin and neurofilament), and often diffuse membranous expression of EMA (Supplementary Fig. 3 [Online Resource 1]; Supplementary Table 3 [Online Resource 2]). Notably, immunostaining for BCOR demonstrated diffuse strong nuclear positivity in two tumors, despite an absence of *BCOR* mutation, tandem duplication, or other gene rearrangement. Meta-analysis of patient outcomes and other clinical data together with previous case reports revealed occurrence during childhood or early adulthood, location most frequently in midline structures (5/7 in the brainstem or spinal cord), and a propensity for local recurrence, with CSF dissemination occurring in two patients leading to mortality (Fig. 1f–h) [9, 10, 12, 14].

**Fig. 1:**
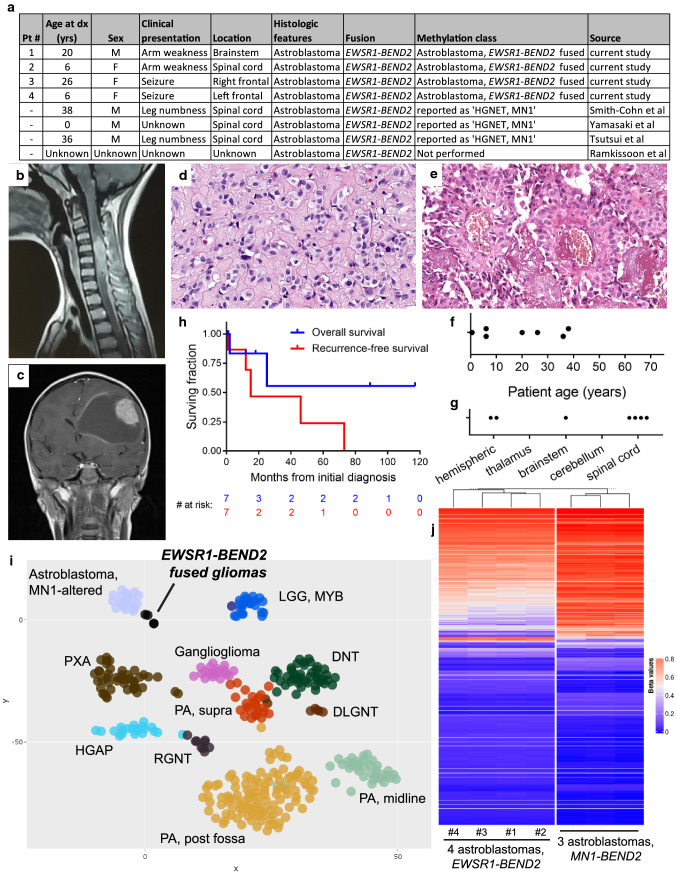
Clinicopathologic features and epigenomic profiling of astroblastoma-like gliomas with *EWSR1-BEND2* fusion. **a** Summary table of the eight patients with gliomas harboring *EWSR1*-*BEND2* fusion, four from this study and four previously reported. **b** MR imaging from patient #2 demonstrating an enhancing mass within the cervical spinal cord. **c** MR imaging from patient #4 demonstrating a solid and cystic mass with an enhancing mural nodule in the left frontal lobe of the brain. **d** Histology from patient #1 showing a glial neoplasm with dense pericellular and perivascular sclerosis. **e** Histology from patient #3 showing astroblastomatous rosettes. **f** Dot plot of patient age at diagnosis for astroblastoma-like gliomas with *EWSR1*-*BEND2* fusion. **g** Dot plot of tumor anatomic location for astroblastoma-like gliomas with *EWSR1*-*BEND2* fusion. **h** Kaplan–Meier plot of overall survival and recurrence-free survival for astroblastoma-like gliomas with *EWSR1*-*BEND2* fusion. **i** tSNE dimensionality reduction plot of genome-wide DNA methylation profiles from the 4 *EWSR1-BEND2* fused gliomas alongside 366 reference tumors spanning 11 CNS tumor entities. **j** Unsupervised hierarchical clustering of DNA methylation data from the 4 astroblastoma-like gliomas with *EWSR1*-*BEND2* fusion and 3 astroblastomas with confirmed *MN1*-*BEND2* fusion, with a heatmap of the 20,000 most differentially CpG sites shown

By targeted next-generation DNA sequencing, all four tumors demonstrated *EWSR1* fusion with *BEND2* as the 3′ partner (Supplementary Fig. 4 [Online Resource 1]; Supplementary Table 4 [Online Resource 2]). In three of the tumors, the gene fusion linked exons 1–8 (codons 1–270) of *EWSR1* with exons 2–14 (codons 9–799) of *BEND2*, whereas the remaining tumor linked exons 1–7 (codons 1–199) of *EWSR1* with exons 4–14 (codons 126–799) of *BEND2*. The *EWSR1-BEND2* fusion was the solitary oncogenic alteration identified in all tumors, with an absence of accompanying alterations involving *IDH1/2*, histone H3 genes, *BRAF*, *NF1*, *PRKCA*, *FGFR1/2/3*, *NTRK1/2/3*, *EGFR*, *PDGFRA*, *MET*, *PIK3CA*, *PIK3R1*, *PTEN*, *CDKN2A*, *TP53*, *TERT* (including promoter region), *ATRX*, *CIC*, *FUBP1*, *MN1*, *ZFTA* (c11orf95), *RELA*, *YAP1*, *MYC*, *MYCN*, *MYB*, and *MYBL1* [6]. The quantity of chromosomal copy number aberrations was variable, but no tumors harbored whole arm co-deletion of chromosomes 1p and 19q, focal amplifications, or homozygous deletions (Supplementary Fig. 5 [Online Resource 1]; Supplementary Table 5 [Online Resource 2]). Genome-wide DNA methylation profiling was performed on the four tumors using Infinium EPIC 850k Beadchips (Illumina) following the manufacturer’s recommended protocols (see Supplementary Methods [Online Resource 3]). tSNE clustering of the DNA methylation data alongside a reference cohort of CNS tumors revealed that the *EWSR1-BEND2* fused gliomas resolved into a single epigenetic group that was divergent from all established reference methylation classes of CNS tumors, but was in close proximity to “High-grade neuroepithelial tumor, *MN1*-altered” (Fig. 1i; Supplementary Fig. 6 [Online Resource 1]; sample manifest in Supplementary Table 6 [Online Resource 2]). Random forest classification using the online DKFZ Brain Tumor Classifier (www.molecularneuropathology.org) demonstrated high-confidence match to the “HGNET, MN1” methylation class for all four tumors (Supplementary Table 7 [Online Resource 2]) [5]. Unsupervised hierarchical clustering successfully segregated the 4 *EWSR1-BEND2* fused gliomas from 3 astroblastomas with confirmed *MN1-BEND2* fusion but revealed highly similar DNA methylation patterns overall (Fig. 1j).

Here we show that gliomas with *EWSR1-BEND2* fusion exhibit astroblastoma-like histologic features and resolve into a single epigenetic subgroup most similar to the methylation class “HGNET, MN1” that encompasses the new tumor type astroblastoma, *MN1*-altered. It remains unclear why *MN1*-*BEND2* and *EWSR1-BEND2* fusions are selected for in astroblastoma, and why *EWSR1* fusion appears to be more common in brainstem and spinal cord astroblastomas relative to *MN1* fusion in hemispheric tumors, but we speculate that these represent two biologically distinct subtypes of astroblastoma which awaits confirmation in future studies. In epigenetically-defined astroblastomas with available sequencing data delineating the fusion partners, only a single tumor with *MN1* fused to a partner other than *BEND2* has been reported to date (dkfz_CNS-PNET_15-0184, GSM1881234 with *MN1*-*CXXC5* fusion) [11]. Including this case series, there are now eight reported astroblastomas with *EWSR1*-*BEND2* fusion (seven with available epigenetic data), indicating that *BEND2* may be a more critical gene for molecular oncogenesis and relevant for tumor classification compared to *MN1*. This is reminiscent of how supratentorial ependymomas were initially defined as being *RELA* fusion positive, but the recognition of a group of supratentorial ependymomas with *ZFTA* (*C11orf95*) fused to other partners besides *RELA* has led to a revised nosology based on *ZFTA* as the defining fusion partner. Thus, these findings support consideration of *BEND2* fusion as the defining molecular alteration for astroblastoma, rather than *MN1* rearrangement as is currently recognized. Future studies are required to define the full biologic and clinicopathologic spectrum of astroblastomas, including establishment of evidence-based grading criteria and determination of optimal treatment regimens.

## Supplementary Information

Below is the link to the electronic supplementary material.

Supplementary file1 (PDF 36835 KB)

Supplementary file2 (XLSX 45 KB)

Supplementary file3 (DOCX 28 KB)

## Data Availability

Digitally scanned image files of representative H&E and immunostained sections from the 4 tumors are available at the following link: https://figshare.com/projects/Astroblastoma-like_gliomas_with_EWSR1-BEND2_fusion/124222. DNA methylation array data files are available from the Gene Expression Omnibus (GEO) repository under accession number GSE183972 (https://www.ncbi.nlm.nih.gov/geo/). Structural variant and copy number data are available in the electronic supplementary material. Raw sequencing data files are available upon request.
