# Supplementary material for: EWSR1-BEND2 fusion defines an epigenetically distinct subtype of astroblastoma
Source: Acta Neuropathol. 2021 Nov 25;143(1):109–13. doi: 10.1007/s00401-021-02388-y (PMC8732961; doi:10.1007/s00401-021-02388-y)
Supplement: Supplementary file 1 — Supplementary file1 (PDF 36835 KB) [file 401_2021_2388_MOESM1_ESM.pdf]

Patient #2
6 y/o F
Astroblastoma
EWSR1-BEND2 fusion

Left: sagittal T1 post-contrast
Right: sagittal T2

Patient #3
26 y/o F
Astroblastoma
EWSR1-BEND2 fusion

Left: sagittal T1 post-contrast
Right: axial T1 post-contrast

Patient #4
6 y/o F
Astroblastoma
EWSR1-BEND2 fusion

Left: coronal T1 post-contrast
Right: axial T1 post-contrast

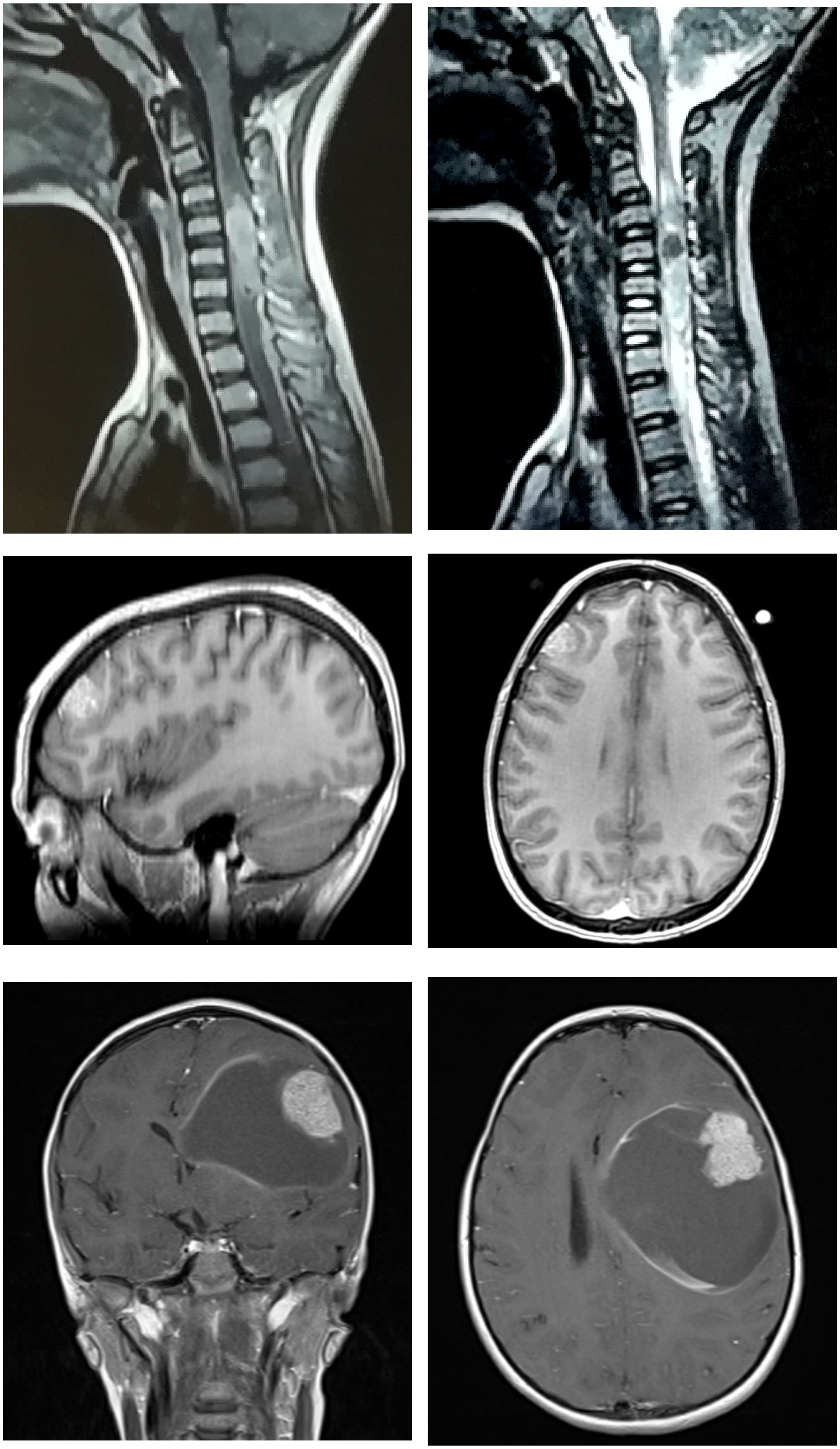

Supplementary Figure 1. Imaging features of *EWSR1-BEND2* fused gliomas.

Patient #1, 20 y/o M, brainstem, *EWSR1-BEND2* fusion, histologic features = astroblastoma

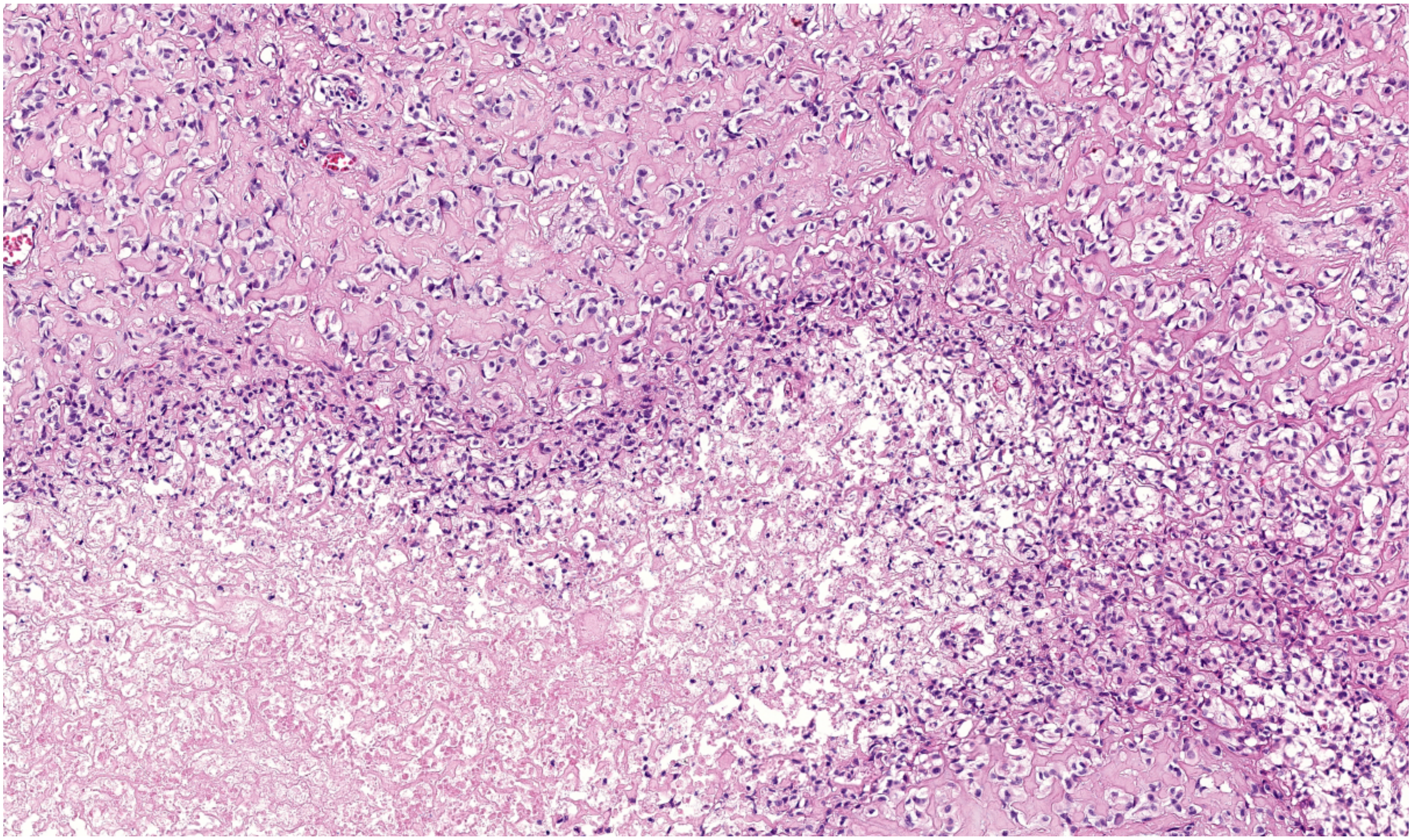

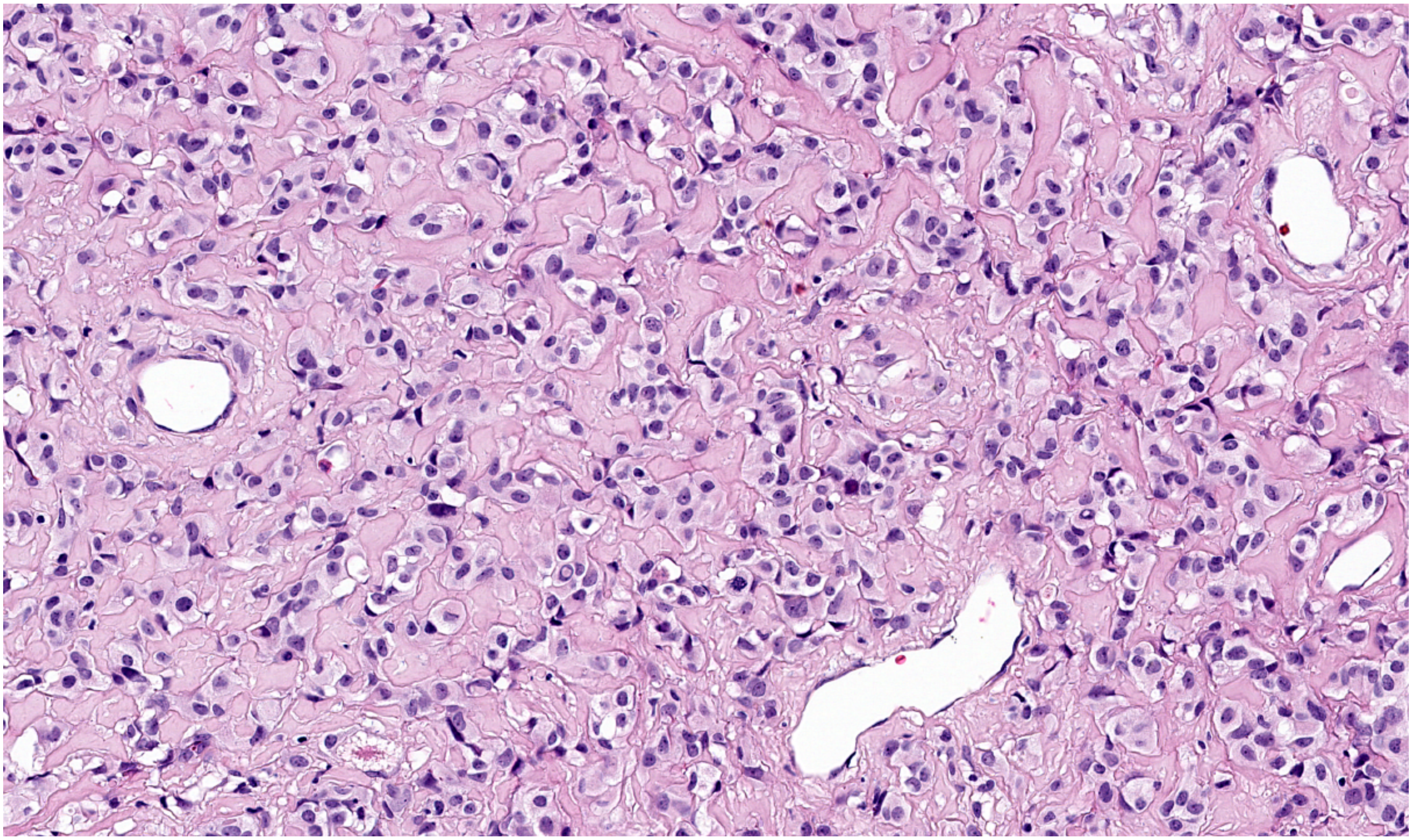

Supplementary Figure 2. Histologic features of *EWSR1-BEND2* fused gliomas. Shown are H&E stained sections at various mangifications for each of the four tumors.

Patient #2, 6 y/o F, cervical spinal cord, *EWSR1-BEND2* fusion, histologic features = astroblastoma

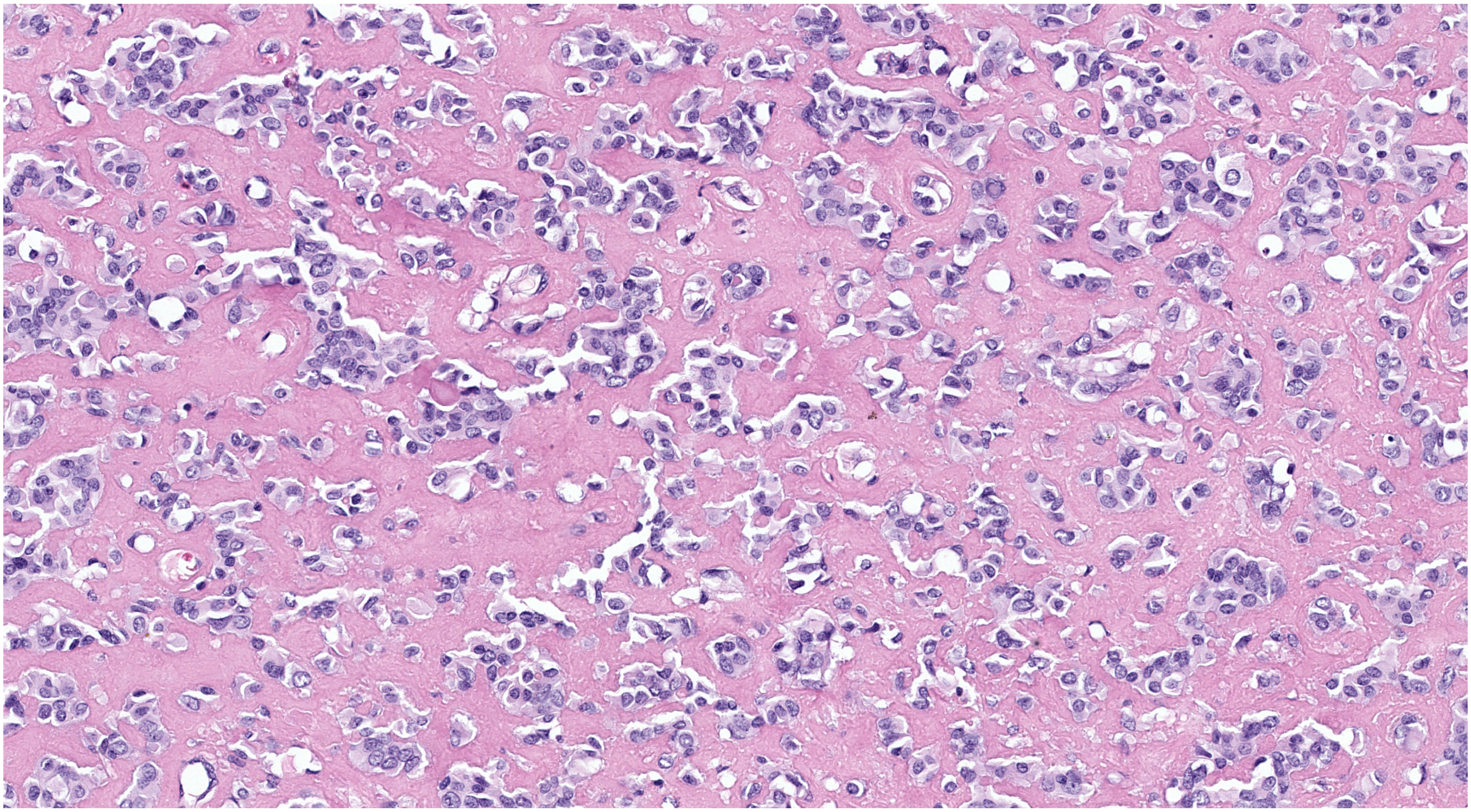

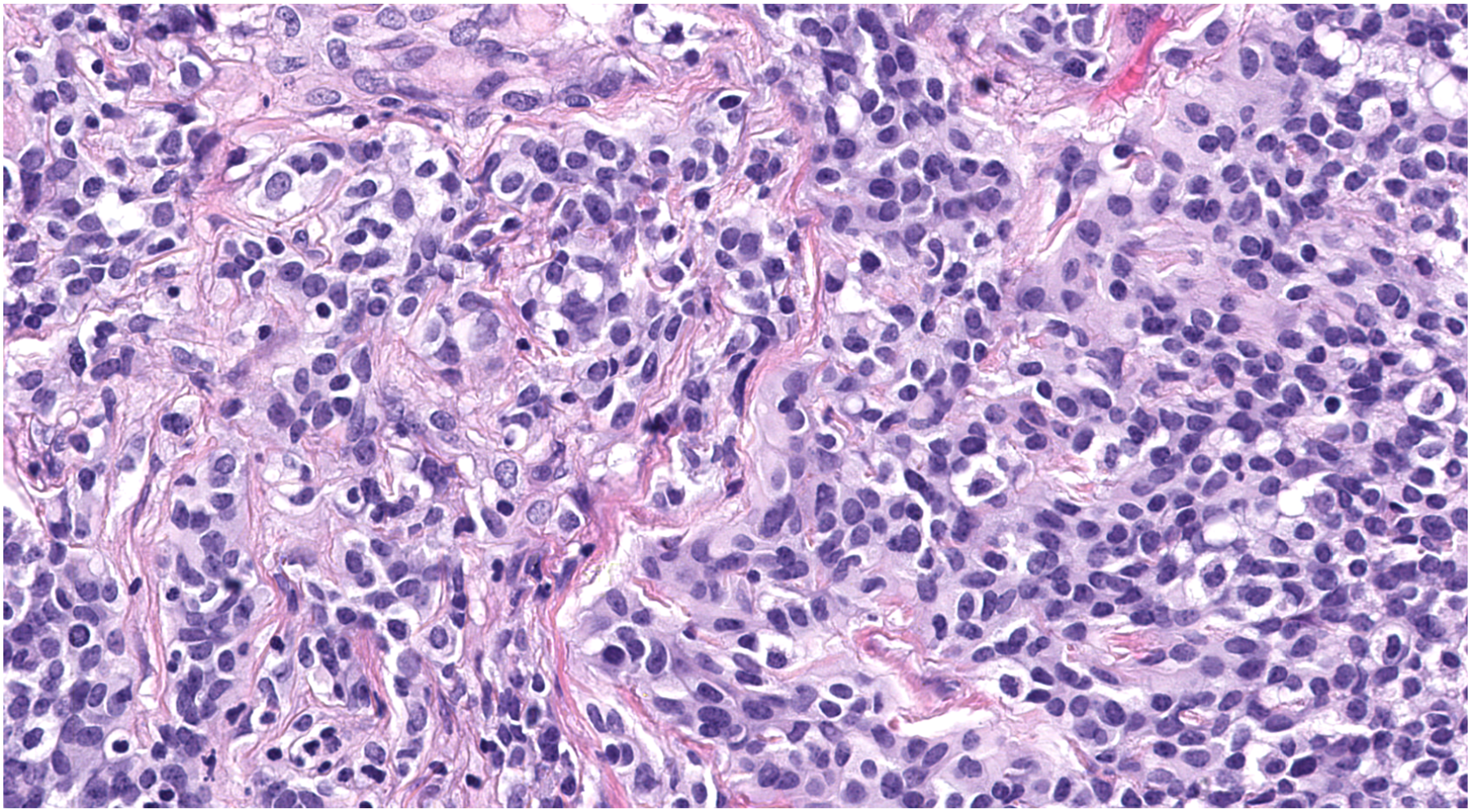

Patient #3, 26 y/o F, initial resection, R frontal lobe, *EWSR1-BEND2* fusion, histologic features = astroblastoma

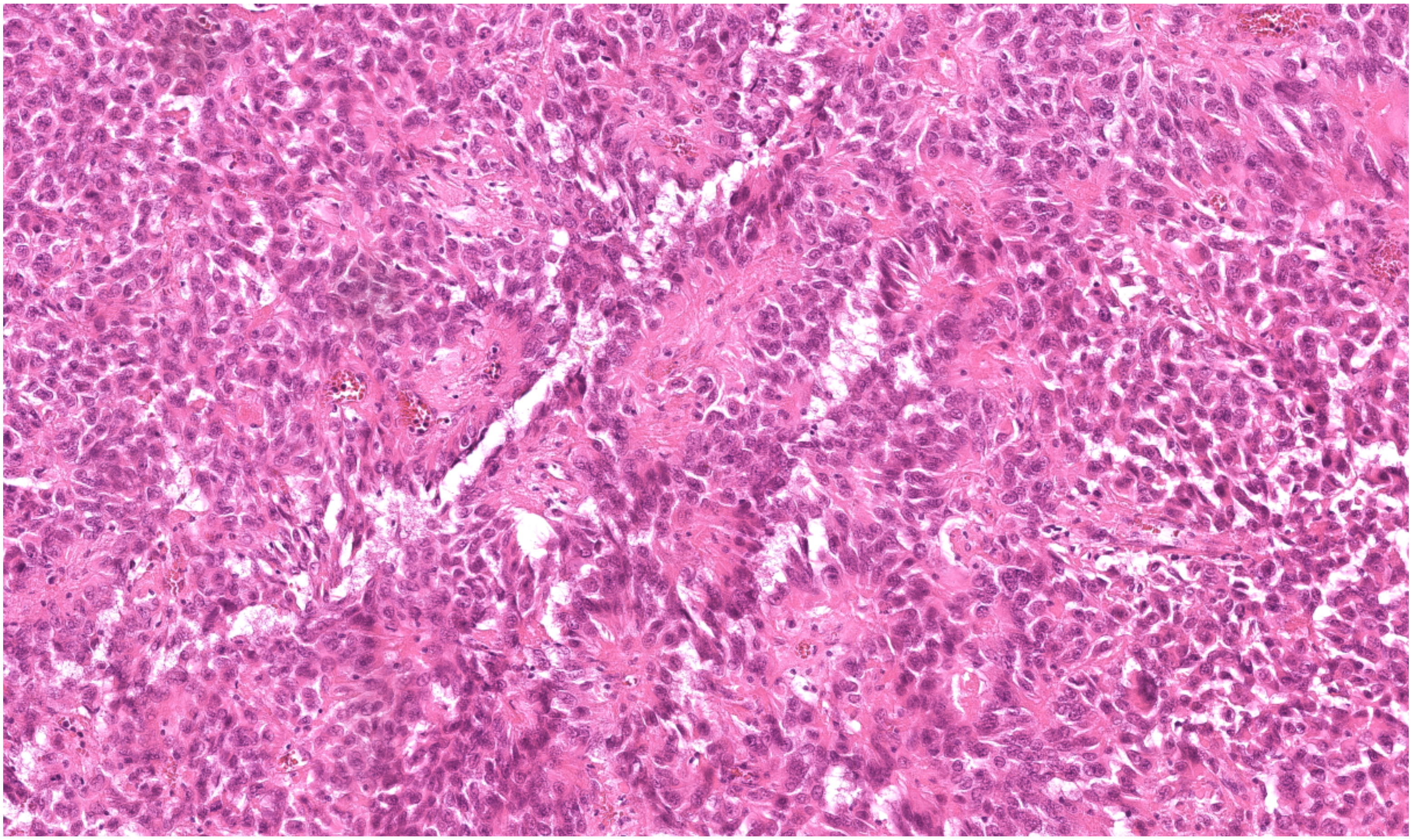

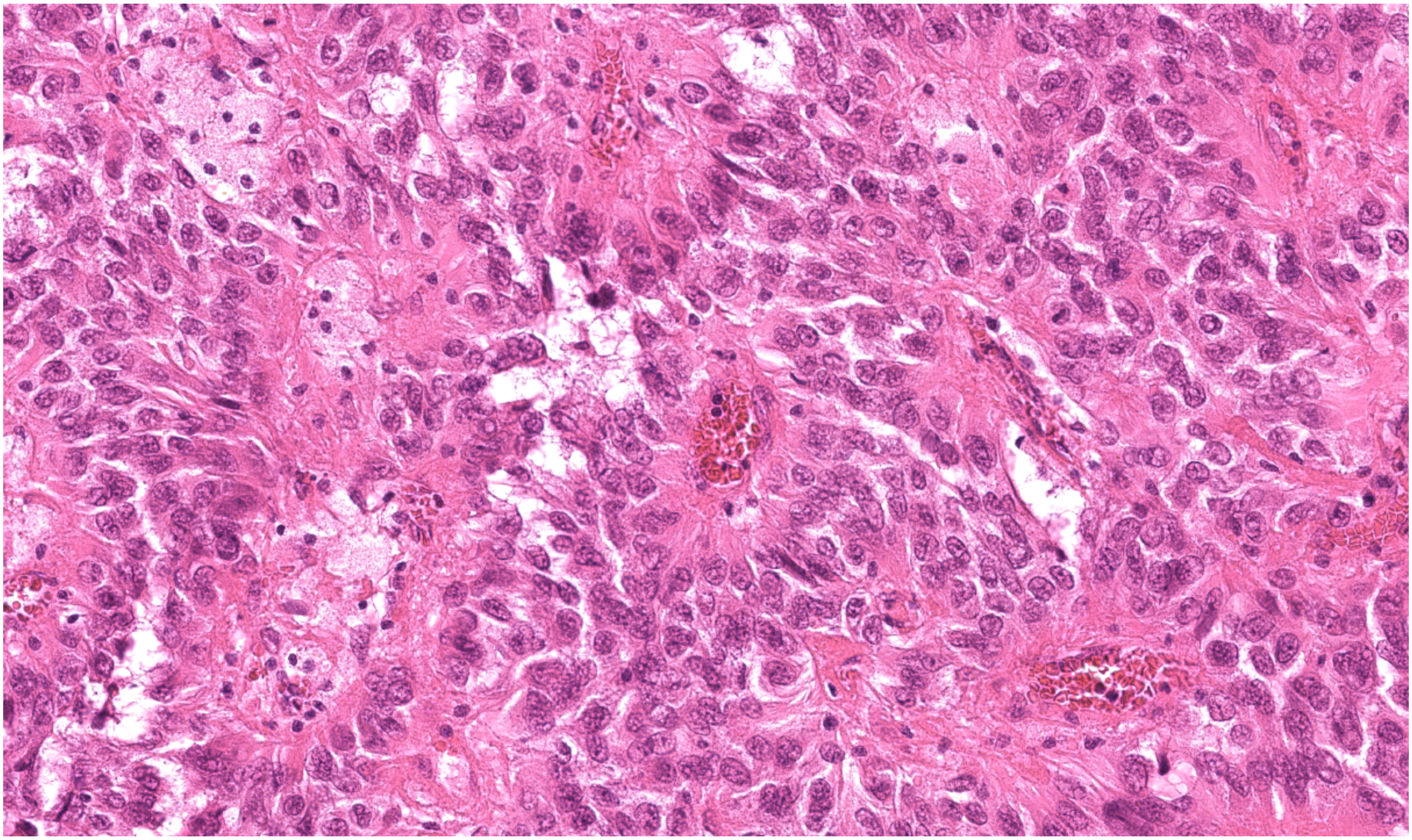

Patient #3, 26 y/o F, recurrent tumor resection, *EWSR1-BEND2* fusion, histologic features = astroblastoma

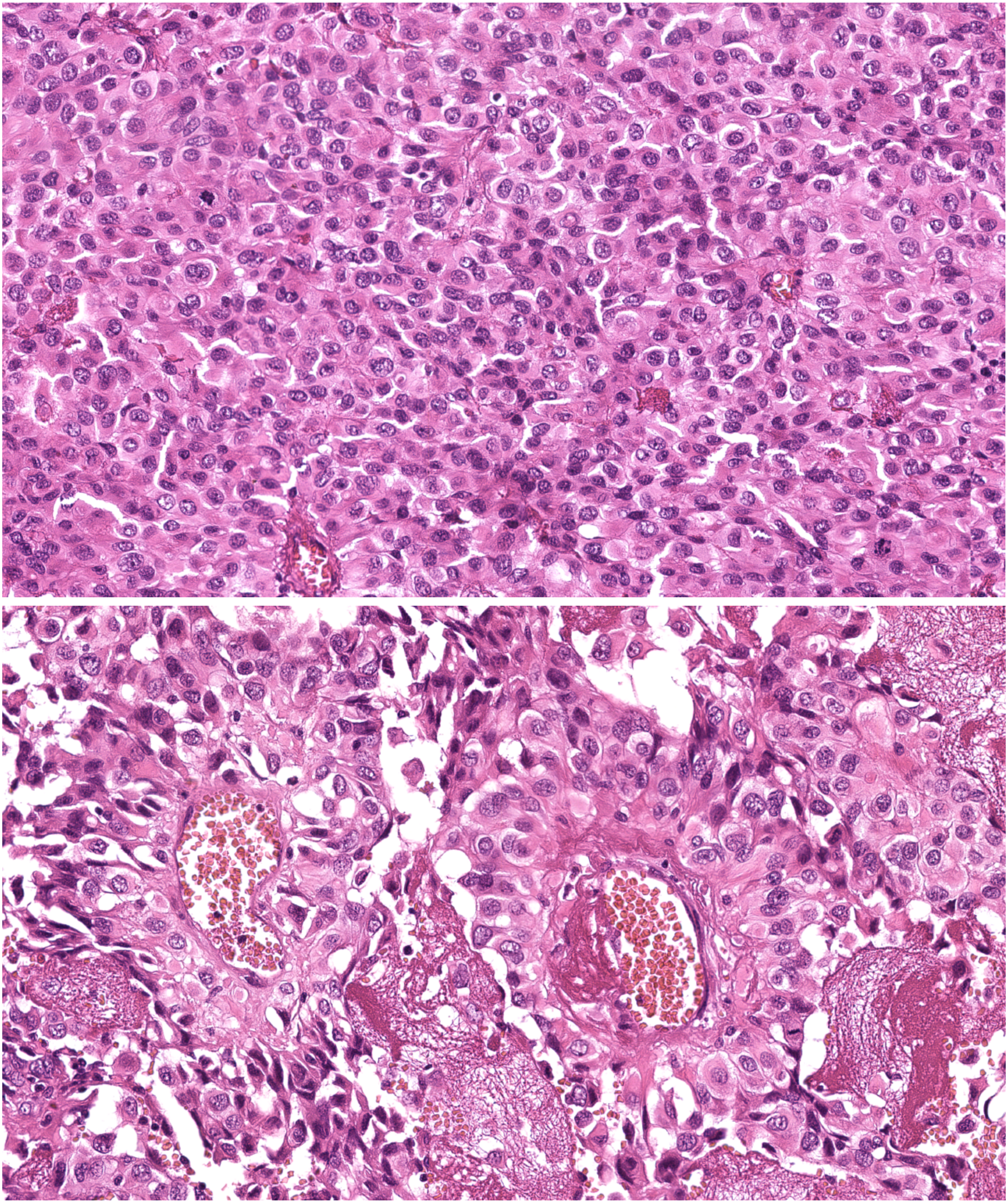

Patient #4, 6 y/o F, left frontal lobe, *EWSR1-BEND2* fusion, histologic features = astroblastoma

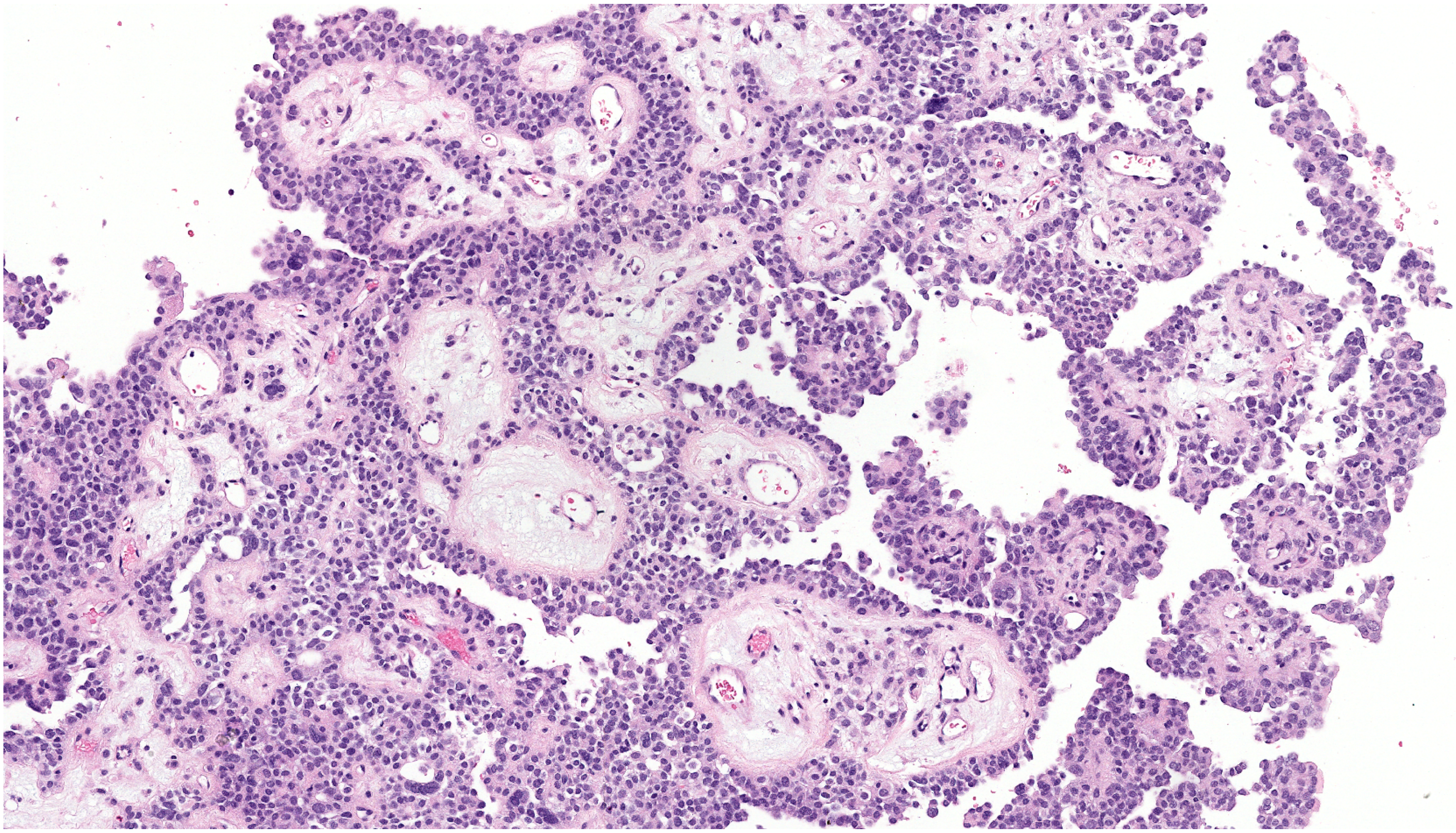

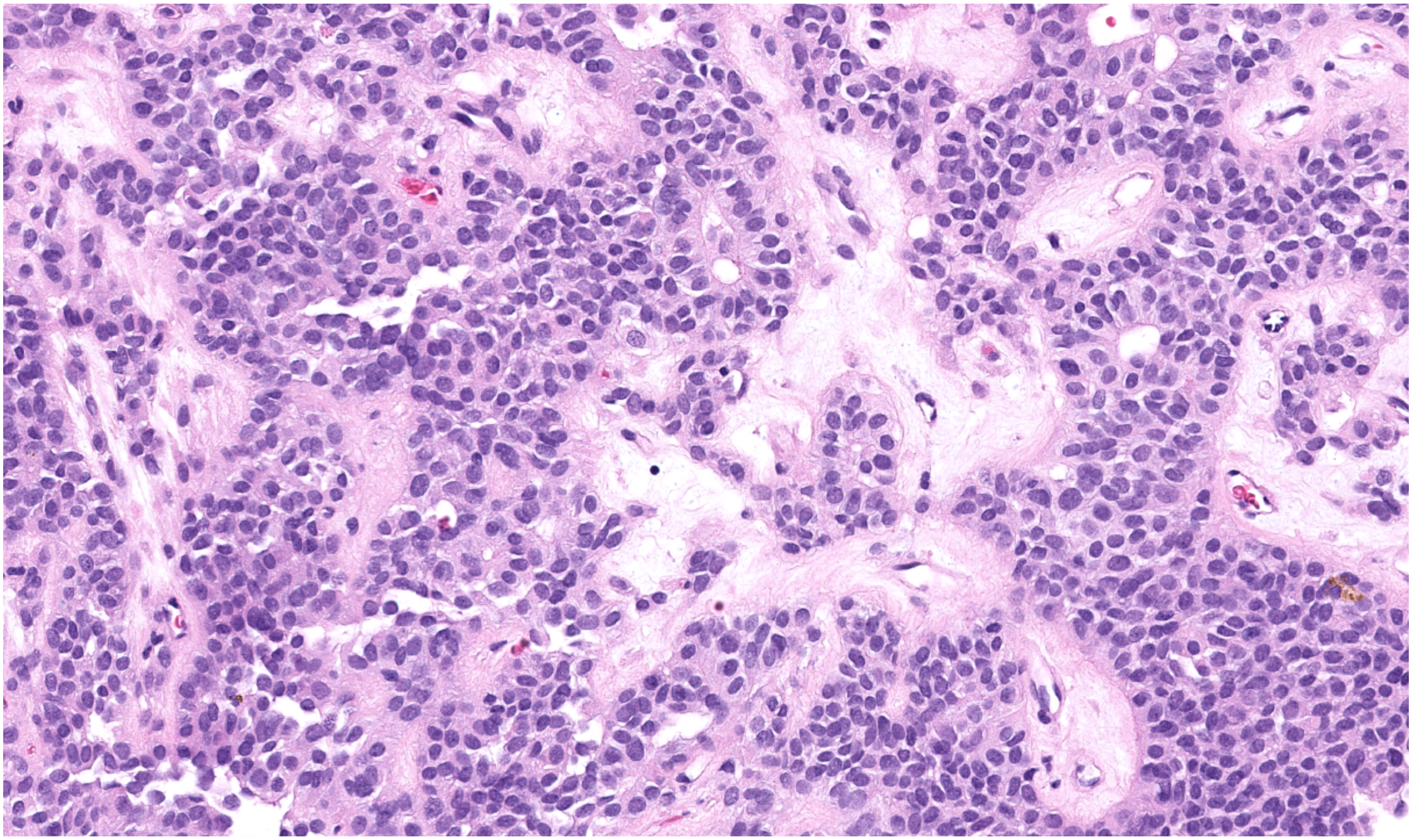

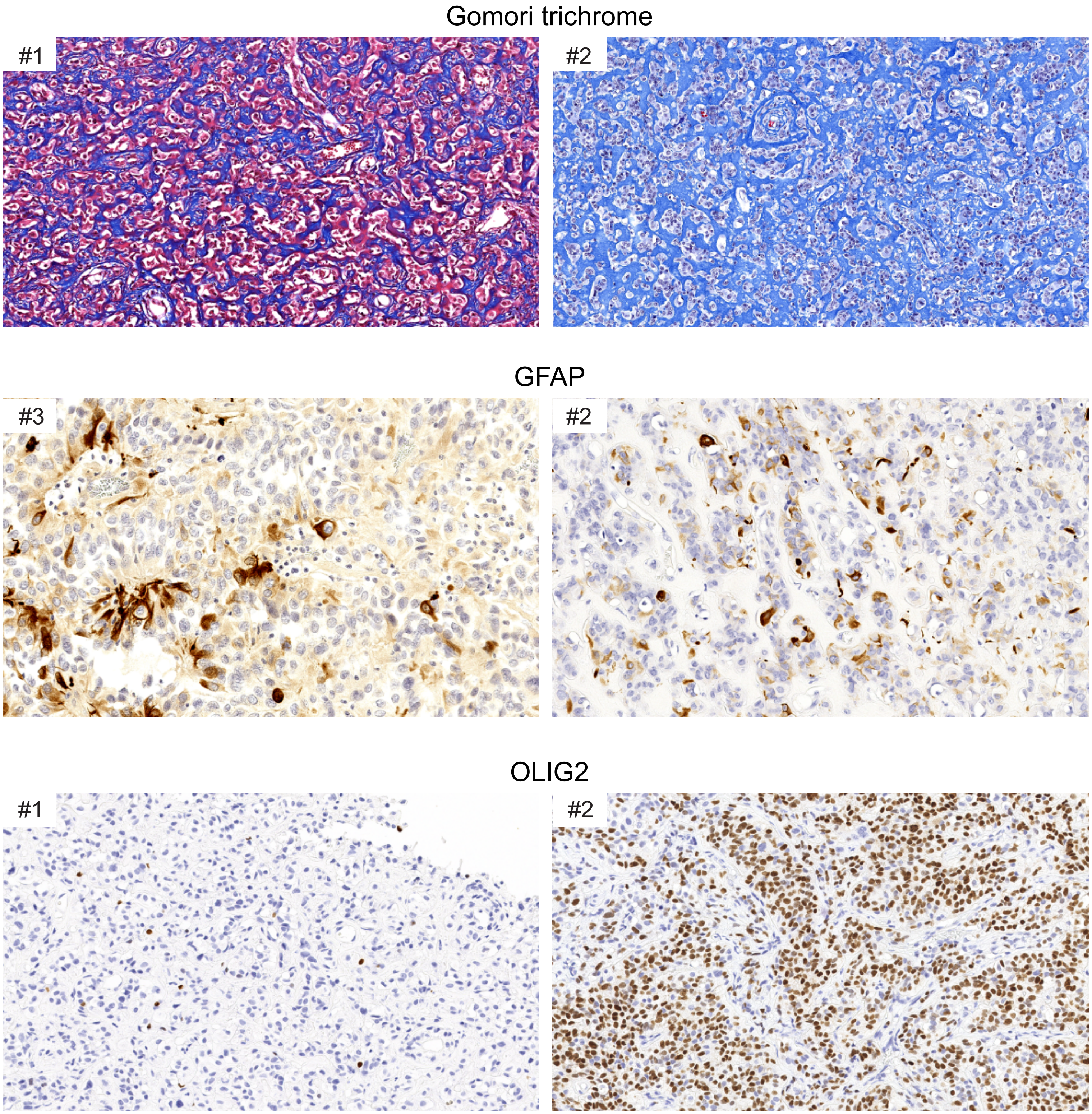

Supplementary Figure 3. Immunohistochemical features of *EWSR1-BEND2* fused gliomas. Shown are representative images of Gomori trichrome staining and immunostains for GFAP, OLIG2, EMA, synaptophysin, and BCOR.

EMA

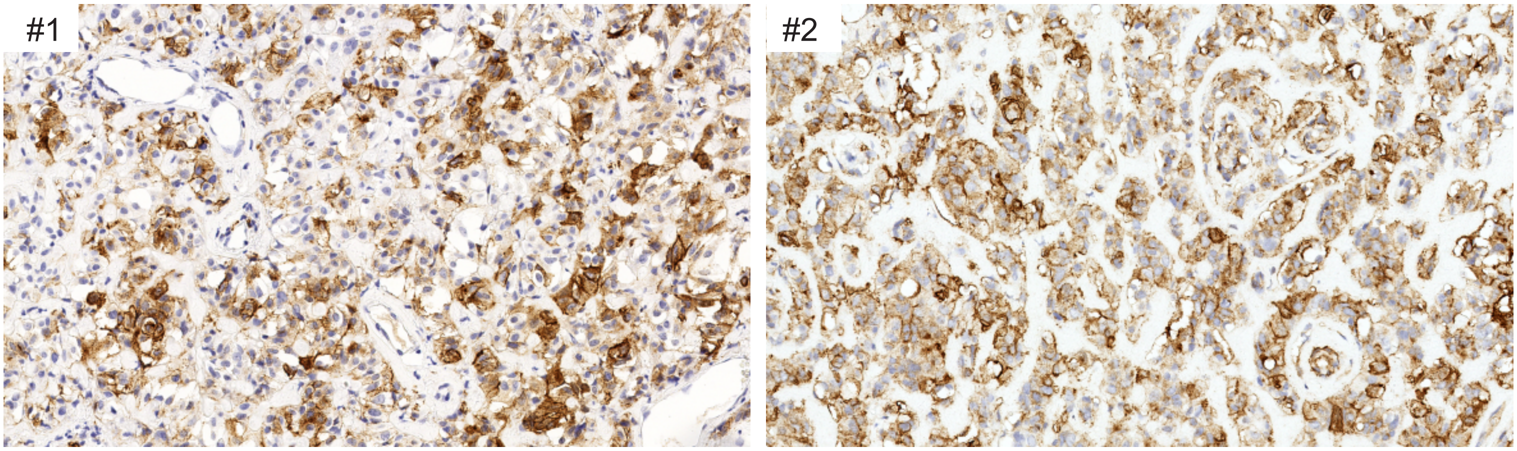

Synaptophysin

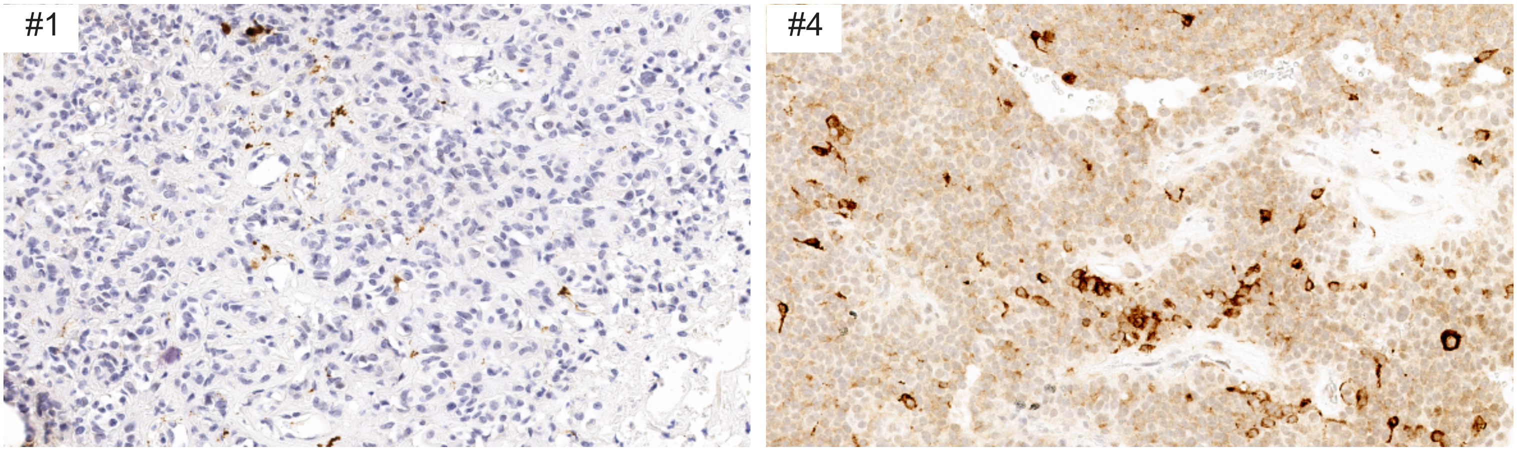

BCOR

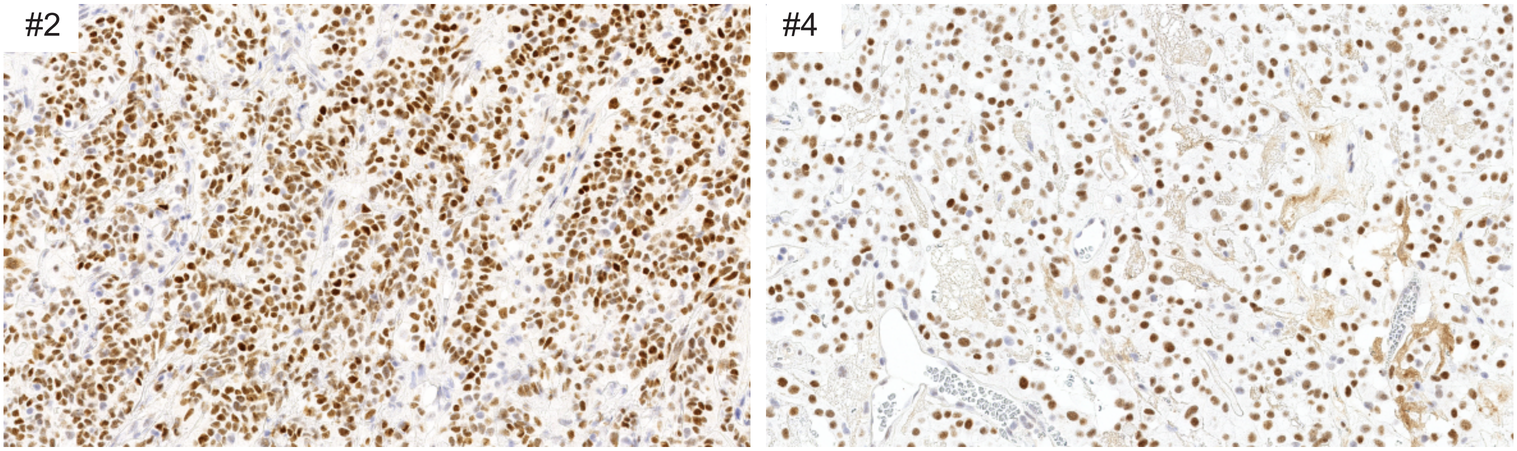

EWSR1-BEND2 fused glioma #4

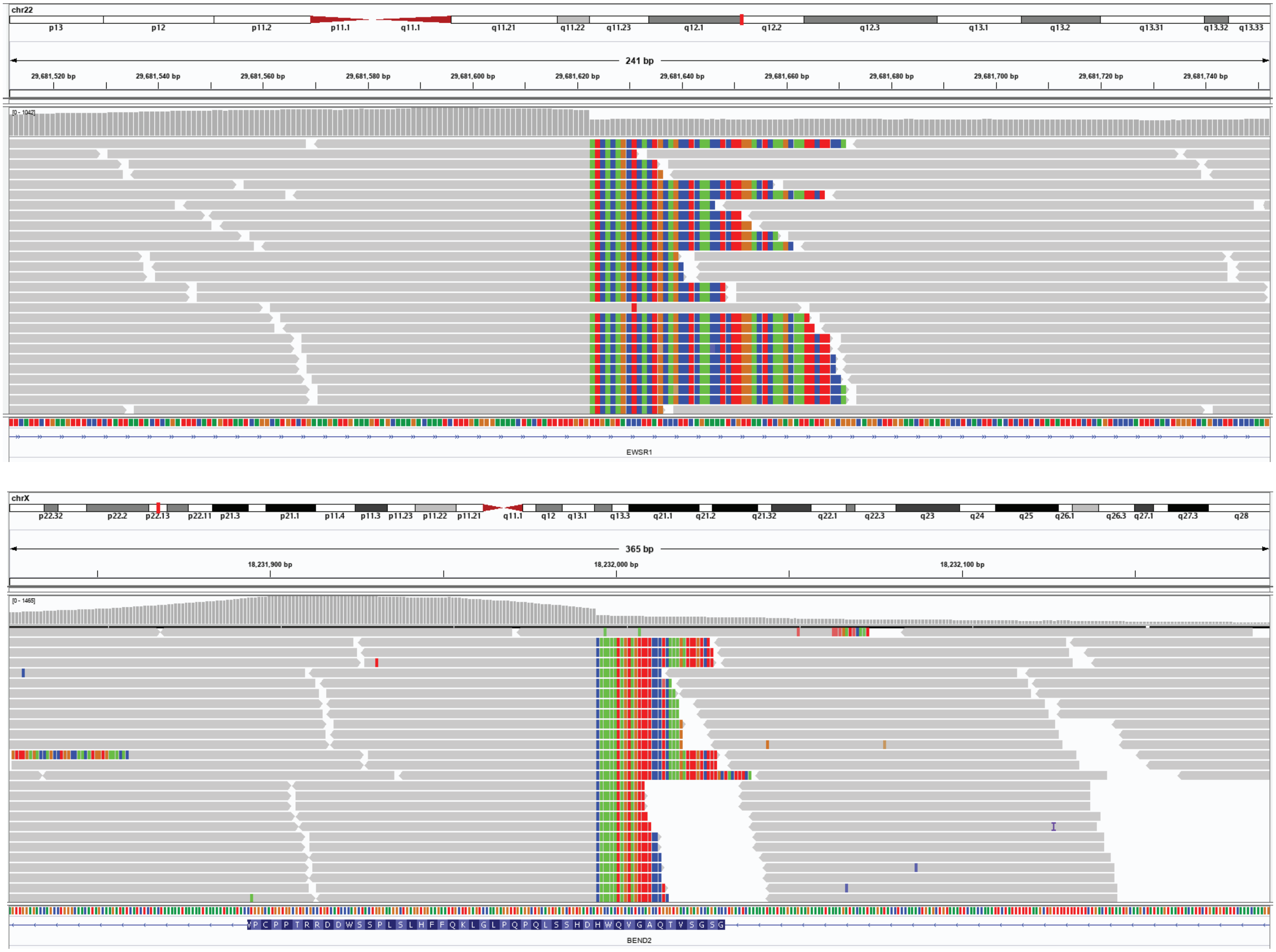

Supplementary Figure 4. Snapshots from the Integrative Genome Viewer showing sequencing reads spanning the *EWSR1-BEND2* fusion breakpoints in tumor #4. The fusion junction is between intron 7-8 of *EWSR1* and exon 3 of *BEND2*. This fusion is expected to result in an in-frame fusion transcript with the 5' portion composed of exons 1-7 (codons 1-199) of *EWSR1* and the 3' portion composed of exons 4-14 (codons 126-799) of *BEND2*. RefSeq transcripts used for annotation: *EWSR1*, NM_013986; *BEND2*, NM_153346.

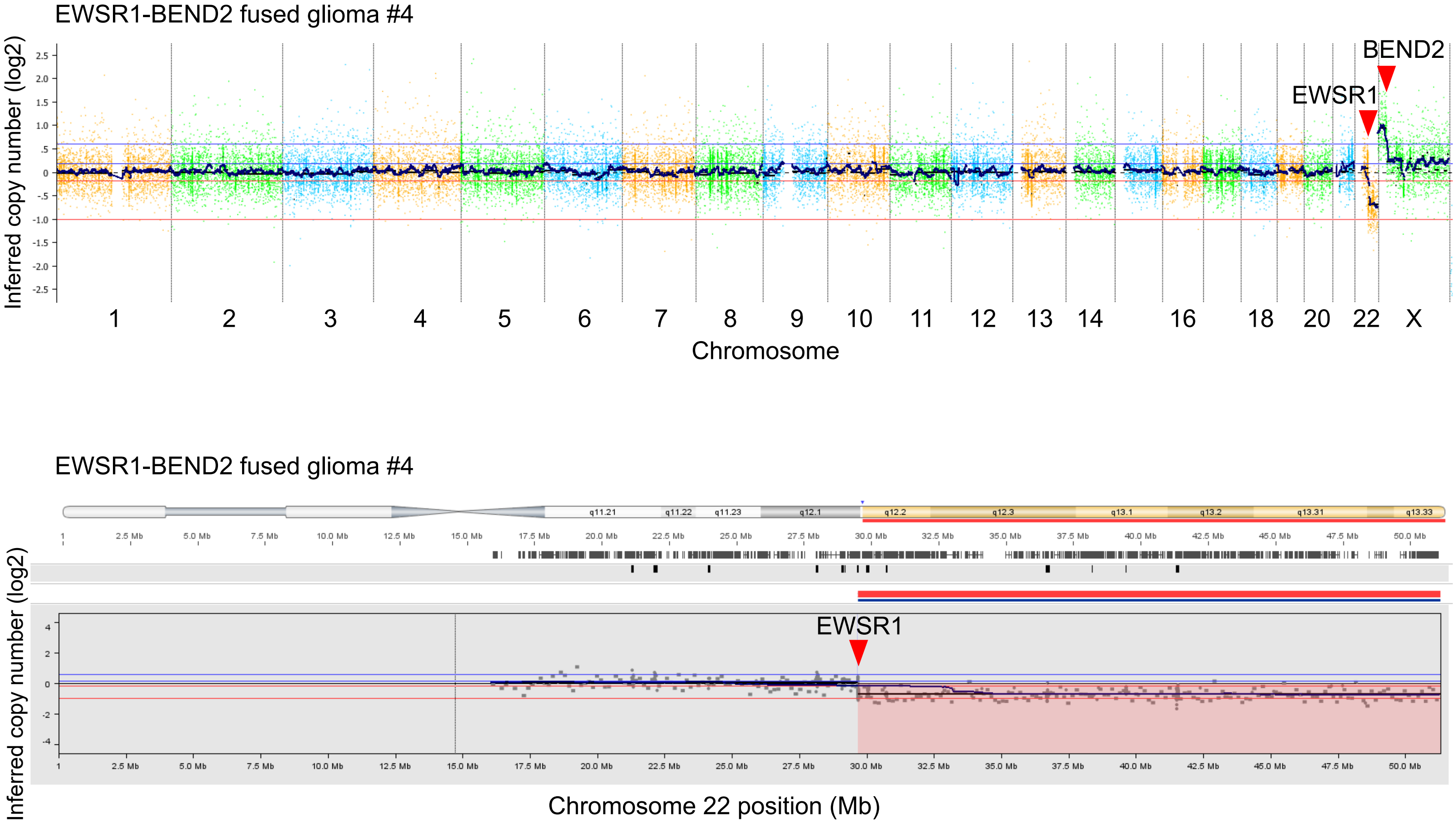

Supplementary Figure 5. Chromosomal copy number plots for tumor #4 demonstrating that the *EWSR1-BEND2* fusion resulted from an unbalanced translocation between the *EWSR1* locus at chromosome 22q12.2 and the *BEND2* locus at chromosome Xp22.13.

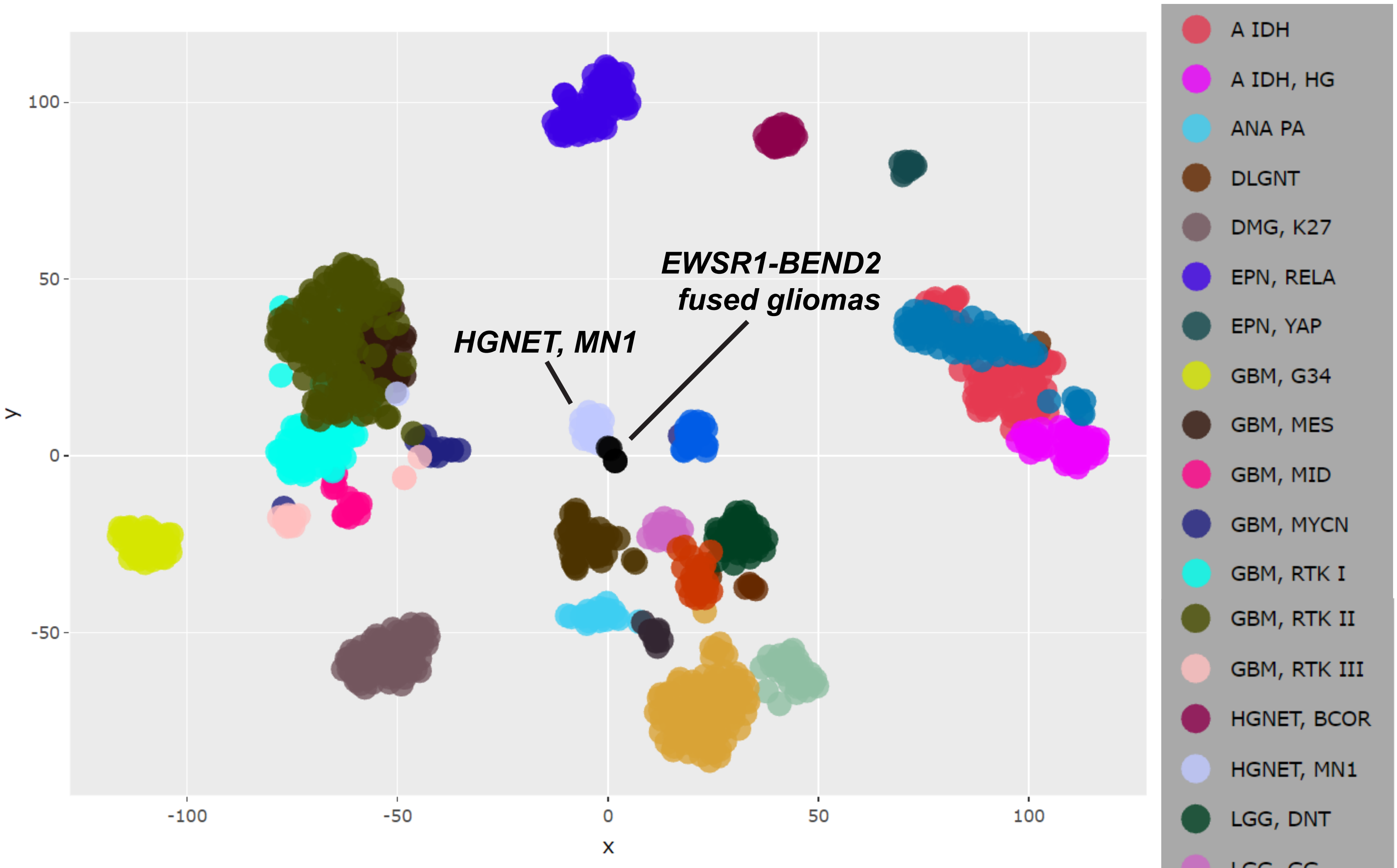

Supplementary Figure 6. Gliomas with *EWSR1-BEND2* fusion resolve into a distinct epigenetic group most similar to the methylation class "HGNET, MN1". tSNE plot of genome-wide DNA methylation profiles from 4 *EWSR1-BEND2*-fused gliomas alongside 1,099 reference tumors spanning 25 CNS tumor entities.

A IDH - astrocytoma, IDH-mutant; A IDH, HG - astrocytoma, IDH-mutant, high-grade; ANA PA - high-grade astrocytoma with piloid features; DLGNT - diffuse leptomeningeal glioneuronal tumor; DMG, K27 - diffuse midline glioma, H3 K27-mutant; EPN, RELA - supratentorial ependymoma, RELA-fused; EPN, YAP - supratentorial ependymoma, YAP1-fused; GBM, G34 - diffuse hemispheric glioma, H3 G34-mutant; GBM, MES - glioblastoma, IDH-wildtype, mesenchymal subclass; GBM, MID - glioblastoma, IDH-wildtype, midline subclass; GBM, MYCN - glioblastoma, IDH-wildtype, MYCN subclass; GBM, RTK I - glioblastoma, IDH-wildtype, RTK1 subclass; GBM, RTK II - glioblastoma, IDH-wildtype, RTK2 subclass; GBM, RTK III - glioblastoma, IDH-wildtype, RTK3 subclass; HGNET, BCOR - CNS tumor with BCOR tandem duplication; HGNET, MN1 - astroblastoma, MN1-altered; LGG, DNT - dysembryoplastic neuroepithelial tumor; LGG, GG - ganglioglioma; LGG, MYB - pediatric-type diffuse low-grade glioma, MYB/MYBL1 fusion positive; LGG, PA MID - pilocytic astrocytoma, midline subclass; LGG, PA PF - pilocytic astrocytoma, posterior fossa subclass; LGG, PA/GG ST - pilocytic astrocytoma, supratentorial subclass; LGG, RGNT - rosette-forming glioneuronal tumor; O IDH - oligodendroglioma, IDH-mutant and 1p/19q-codeleted; PXA - pleomorphic xanthoastrocytoma
