# Supplementary material for: EWSR1-BEND2 fusion defines an epigenetically distinct subtype of astroblastoma
Source: Acta Neuropathol. 2021 Nov 25;143(1):109–13. doi: 10.1007/s00401-021-02388-y (PMC8732961; doi:10.1007/s00401-021-02388-y)
Supplement: Supplementary file 3 — Supplementary file3 (DOCX 28 KB) [file 401_2021_2388_MOESM3_ESM.docx]

**Online Resource 3.** Supplementary Methods.

*Patient cohort and tumor samples*

Four patients with gliomas harboring *EWSR1-BEND2* fusion identified by capture-based next-generation DNA sequencing were included in this study. All tumor specimens were fixed in 10% neutral-buffered formalin and embedded in paraffin. Tumor tissue was selectively scraped from unstained slides or punched from formalin-fixed, paraffin-embedded blocks using 2.0 mm disposable biopsy punches (Integra Miltex Instruments, cat# 33-31-P/25) to enrich for as high of tumor content as possible. Genomic DNA was extracted from this macrodissected formalin-fixed, paraffin-embedded tumor tissue using the QIAamp DNA FFPE Tissue Kit (Qiagen).

*Targeted next-generation DNA sequencing*

Targeted next-generation DNA sequencing was performed using the UCSF500 NGS Panel as previously described [Kline et al]. Capture-based next-generation DNA sequencing was performed using an assay that targets all coding exons of 479 cancer-related genes, select introns and upstream regulatory regions of 47 genes to enable detection of structural variants including gene fusions, and DNA segments at regular intervals along each chromosome to enable genome-wide copy number and zygosity analysis, with a total sequencing footprint of 2.8 Mb. Multiplex library preparation was performed using the KAPA Hyper Prep Kit (Roche) according to the manufacturer’s specifications using 250 ng of sample DNA. Hybrid capture of pooled libraries was performed using a custom oligonucleotide library (Nimblegen SeqCap EZ Choice). Captured libraries were sequenced as paired-end 100 bp reads on an Illumina HiSeq 2500 instrument. Sequence reads were mapped to the reference human genome build GRCh37 (hg19) using the Burrows-Wheeler aligner (BWA). Recalibration and deduplication of reads was performed using the Genome Analysis Toolkit (GATK). Coverage and sequencing statistics were determined using Picard CalculateHsMetrics and Picard CollectInsertSizeMetrics. Single nucleotide variant and small insertion/deletion mutation calling was performed with FreeBayes, Unified Genotyper, and Pindel. Large insertion/deletion and structural alteration calling was performed with Delly. Variant annotation was performed with Annovar. Single nucleotide variants, insertions/deletions, and structural variants were visualized and verified using Integrative Genome Viewer. Genome-wide copy number and zygosity analysis was performed by CNVkit and visualized using Nexus Copy Number (Biodiscovery).

*DNA methylation profiling*

Genomic DNA from 4 gliomas harboring *EWSR1-BEND2* fusion was bisulfite converted using the EZ DNA Methylation kit following the manufacturer’s recommended protocol (Zymo Research). Bisulfite converted DNA was then amplified, fragmented, and hybridized to Infinium EPIC 850k Human DNA Methylation BeadChips following the manufacturer’s recommended protocol (Illumina). Methylation data were preprocessed using the minfi package (v1.30.0) in R Bioconductor (version 3.5.3) [Aryee et al]. The detection p-value for each sample was computed, and CpG sites with detection p values above 0.05 were discarded from the analysis. Additional quality control was performed by calculating the median log (base2) intensities for methylated and unmethylated signals for each array. All samples had unmethylated and methylated median intensity values above 10 that were used for analysis. Functional normalization with NOOB background correction and dye-bias normalization was performed [Fortin et al; Triche et al]. Probe filtering was performed after normalization. Specifically, probes located on sex chromosomes, containing nucleotide polymorphisms (dbSNP132 Common) within five base pairs of and including the targeted CpG site, or mapping to multiple sites on hg19 (allowing for one mismatch), as well as cross reactive probes were removed from analysis.

The DNA methylation profiles of the 4 tumors were assessed together with 1099 reference tumors spanning 25 CNS tumor entities previously generated at DKFZ [Capper et al]. These included 78 A IDH (astrocytoma, IDH-mutant), 46 A IDH-HG (astrocytoma, IDH-mutant, high-grade), 21 ANA PA (anaplastic astrocytoma with piloid features), 8 DLGNT (diffuse leptomeningeal glioneuronal tumor), 78 DMG-K27 (diffuse midline glioma, H3 K27M-mutant), 70 EPN RELA (ependymoma, RELA-fused), 11 EPN YAP (ependymoma, YAP-fused), 41 GBM G34 (diffuse hemispheric glioma, H3 G34-mutant), 56 GBM MES (glioblastoma, IDH-wildtype, mesenchymal subclass), 14 GBM MID (glioblastoma, IDH-wildtype, midline subclass), 16 GBM MYCN (glioblastoma, IDH-wildtype, MYCN subclass), 64 GBM RTK1 (glioblastoma, IDH-wildtype, RTK1 subclass), 143 GBM RTK2 (glioblastoma, IDH-wildtype, RTK2 subclass), 13 GBM RTK3 (glioblastoma, IDH-wildtype, RTK3 subclass), 23 HGNET BCOR (high-grade neuroepithelial tumor, BCOR-altered), 21 HGNET MN1 (high-grade neuroepithelial tumor, MN1-altered), 44 DNT (dysembryoplastic neuroepithelial tumor), 21 GG (ganglioglioma), 22 LGG MYB (low-grade glioma, MYB/MYBL1 fusion positive), 38 PA MID (pilocytic astrocytoma, midline subclass), 114 PA PF (pilocytic astrocytoma, posterior fossa subclass), 24 PA ST (pilocytic astrocytoma, supratentorial subclass), 9 RGNT (rosette-forming glioneuronal tumor), 80 O IDH (oligodendroglioma, IDH-mutant and 1p/19q-codeleted), and 44 PXA (pleomorphic xanthoastrocytoma). Since the reference cohort contained methylation data generated using the Infinium Human Methylation 450k BeadChips, the approximately 450,000 overlapping CpG sites between the EPIC 850k and 450k BeadChips were used in the analysis. A beta value matrix with 403,783 CpG probes was used for all downstream analysis. Row-wise standard deviation was calculated for each probe across all samples, and the 30,000 most differentially methylated probes were selected. Dimensionality reduction using t-distributed stochastic neighbor embedding (t-SNE) was performed by *Rtsne* (v0.15) using the following analysis parameters: dims=2, max_iter=5000, pca=F, theta=0, perplexity=10, eta=200. The tSNE plot was visualized with ggplot2 (v3.2.0) [<https://ggplot2.tidyverse.org/>].

We performed unsupervised hierarchical clustering with the hclust function in Rstats (v3.6.0) to assess variation in DNA methylation patterns among the 4 astroblastoma-like gliomas with *EWSR1*-*BEND2* fusion from this study alongside 3 astroblastomas with confirmed *MN1*-*BEND2* fusion previously generated at DKFZ: dkfz_ABM_15-0004 (9007217139_R06C01, GSM1903728); dkfz_CNS-PNET_15-0280 (9283265033_R03C02, GSM1903468); dkfz_EPN_15-0032 (9426020061_R02C01, GSM1903751) [Sturm et al]. The lmFit function from the Limma package (v.3.40.6) was applied on a log-transformed b-value matrix to identify the 20,000 most differentially methylated CpG probes across the 7 tumors. Then K-means clustering utilizing the Pearson distance matrix with complete linkage was used to determine the optimal number of clusters, through 500 re-sampling interactions of the dataset for K-means of 2, 3, 4, or 5. Visualization was performed using the R package ComplexHeatmap (v2.0.0) [Gu et al].

**References for Supplementary Methods**

Aryee MJ, Jaffe AE, Corrada-Bravo H et al (2014) Minfi: a flexible and comprehensive Bioconductor package for the analysis of Infinium DNA methylation microarrays. Bioinformatics 30:1363-1369

Capper D, Jones DTW, Sill M et al (2018) DNA methylation-based classification of central nervous system tumours. Nature 555:469-474

Fortin JP, Labbe A, Lemire M et al (2014) Functional normalization of 450k methylation array data improves replication in large cancer studies. Genome Biol 15:503

Gu Z, Eils R, Schlesner M (2016) Complex heatmaps reveal patterns and correlations in multidimensional genomic data. Bioinformatics 32:2847-2849

Kline CN, Joseph NM, Grenert JP et al (2017) Targeted next-generation sequencing of pediatric neuro-oncology patients improves diagnosis, identifies pathogenic germline mutations, and directs targeted therapy. Neuro Oncol 19:699-709

Sturm D, Orr BA, Toprak UH et al (2016) New brain tumor entities emerge from molecular classification of CNS-PNETs. Cell 164:1060-1072

Triche TJ, Weisenberger DJ, Van Den Berg D, Laird PW, Siegmund KD (2013) Low-level processing of Illumina Infinium DNA Methylation BeadArrays. Nucleic Acids Res 41:e90
